# Antimicrobial and Anti-Biofilm Activities of *Thymus fallax* Essential Oil against Oral Pathogens

**DOI:** 10.1155/2022/9744153

**Published:** 2022-09-12

**Authors:** Maryam Moshaverinia, Sarina Sahmeddini, Fatemeh Lavee, Zahra Zareshahrabadi, Kamiar Zomorodian

**Affiliations:** ^1^Department of Oral and Maxillofacial Disease, School of Dentistry, Shiraz University of Medical Sciences, Shiraz, Iran; ^2^Student Research Committee, Shiraz University of Medical Sciences, Shiraz, Iran; ^3^Oral and Dental Disease Research Center, Oral and Maxillofacial Disease Department, School of Dentistry, Shiraz University of Medical Sciences, Shiraz, Iran; ^4^Basic Sciences in Infectious Diseases Research Center, Shiraz University of Medical Sciences, Shiraz, Iran; ^5^Department of Medical Mycology and Parasitology, School of Medicine, Shiraz University of Medical Sciences, Shiraz, Iran

## Abstract

**Aim:**

Oral infections associated with a wide diversity of microorganisms, including bacteria and yeasts, occur frequently in humans, affecting the whole oral cavity and well-being. Oral pathogens easily grow and propagate in the oral cavity, leading to the formation of dental plaque on both soft and hard tissue. The oral cavity contains up to 700 different species of microorganisms, which *Candida* and *Streptococci* are the most common organisms. Oral diseases continue to increase despite the best efforts of the medical and scientific communities. During the past decades, drug resistance to common antibiotics used in the treatment of oral infections has been raised to high levels worldwide. To overcome such resistance, there is a growing tendency to use herbal medicine as alternative. This study was conducted to find out the chemical constitution of *Thymus fallax* (*T. fallax*) essential oil and to determine its antimicrobial and anti-biofilm efficacy against common oral pathogens.

**Materials and Methods:**

The chemical compositions of the essential oil distilled from *T. fallax* plants were analyzed using gas chromatography/mass spectrometry (GC/MS). Antimicrobial susceptibility testing against common *Streptococcus*, *Enterococcus*, *Staphylococcus*, and *Candida* strains was assessed by broth microdilution in 96-well plates as suggested by the Clinical and Laboratory Standards Institute (CLSI) methods. Biofilm growth and development were assessed using XTT reduction assay.

**Results:**

Based on the GC/MS test results, *thymol* (67.75%) followed by *caryophyllene* (E-) (7.27%) was the main component of this essential oil*. T. fallax* inhibited the growth of examined microbial pathogens at concentrations of 0.031-16 *μ*L/mL. Also, the essential oil showed biofilm inhibition of greater than 95% in the concentration of 8 *μ*L/mL against all tested bacterial strains as well as *Candida albicans* (*p* value < 0.05).

**Conclusions:**

Considering the significant antimicrobial activities of *T. fallax*, this essential oil has the potential to be used as further antimicrobial and anti-biofilm pharmaceutical products in the control and treatment of oral infections.

## 1. Introduction

Oral infections caused by microorganisms are one of the most common infections worldwide [1, 2]. It is now well known that oral infections can have different impacts on people's well-being and life quality, causing pain, discomfort, and affecting proper physical functions [3, 4]. Besides, oral health is also associated with some sorts of systemic diseases such as diabetes, cardiovascular, and respiratory diseases [5]. Therefore, balancing the oral microflora and preventing the overgrowth of pathogens in the oral cavity is very important to improving oral health. Oral bacteria and *Candida albicans* are highly associated with oral diseases and infections, including dental caries, periodontitis, glossitis, and mucositis. Although some of them are normal flora of the oral cavity, growth and pathogenesis can be accelerated in patients with immune deficiencies [2, 6–8].

Currently, *Streptococcus* strains such as *S. mutans* and *S. sobrinus* are considered the most common bacteria isolated from dental plaque biofilms [9, 10]. *Staphylococci* are also abundant in dental implant and denture surfaces and supragingival and subgingival biofilm and plaque [11, 12] *Staphylococcus* contamination during implant surgery and its subsequent adhesion are still the primary causes of implant failure [11]. *Enterococcus faecalis*, another component of the oral microbiota, is mainly involved in failures of endodontic treatments, causing oral infections. Evaluation of *Enterococcus faecalis* biofilm formation by microtiter plate method presented 49% of the isolates as strong biofilm producer [13]. Uniquely, *C. albicans* is well adapted to humans' oral cavity; however, any changes in the host environment provide this pathogen the opportunity to invade. Also, failure of antifungal therapies is very common in patients with denture stomatitis since its biofilm is very resistant [8].

For a long time, people used aromatic plants for their medicinal properties [14]. Numerous of these plants and their aromatic products have antimicrobial effects owing to their secondary metabolites like terpenoids [15]. A previous study also demonstrated the usage of essential oil (EO) in the treatment of oral infections [16]. *Thymus* spp., one of the main genera of the *Lamiaceae* family, have shown activity against bacteria and fungi. The EO of *T. fallax* also showed antibacterial activity against some other bacteria in the gastrointestinal tract [17].

Due to the high incidence of oral infections, increased resistance to common antibiotics, and the adverse effects of some chemical medications currently used in dentistry (e.g., nausea, diarrhea, vomiting, and tooth staining) [18], there is a great need for an alternative approach and novel compounds. Therefore, in this study, our aim is to investigate the *in vitro* antimicrobial and anti-biofilm efficacy of *T. fallax* EOs against oral pathogens.

## 2. Method and Material

### 2.1. *T. fallax* Extraction and Analyzation

*T. fallax* was harvested locally in the west of Iran and was air dried under proper conditions. Plant and voucher specimen were deposited in the herbarium of the Shiraz University of Medical Sciences. The aerial parts of the plant were powdered by an automatic blender and were hydrodistillated by all glass Clevenger type apparatus for 48 hours. Hydrous sodium sulfate was then added for dehumidification. The oil was restored in a sealed vial at a temperature of -20C until GC (gas chromatography) and GC-MS (gas chromatography-mass spectrometry) were done. Compounds were identified by comparing their mass spectra fragmentation pattern and retention time with standard reference compounds and comparing with retention times of n-alkanes (C_6_-C_24_) [19].

### 2.2. Microorganisms

Microorganisms were obtained from the Department of Microbiology, Shiraz University of Medical Sciences, Shiraz, Iran.

In this study, standard strains of *Streptococcus mutans* (ATCC35668), *Streptococcus sanguinis* (ATCC10556), *Streptococcus salivarius* (ATCC9222), *Streptococcus sobrinus* (ATCC27607), *Staphylococcus aureus* (ATCC52268), *Enterococcus faecalis* (ATCC51299), and *Candida albicans* (ATCC 10261) were used.

### 2.3. Determination of Minimum Inhibitory Concentration

The minimum inhibitory concentrations (MIC) were determined using the broth microdilution method; the inocula of bacteria and yeasts were prepared from 24-hour broth cultures. Suspensions were adjusted to 0.5 McFarland standard turbidity, that is, a stock suspension of 1 − 1.5 × 10^8^ cells/mL for bacteria and 1–5 × 10^6^ cells/mL for yeasts. For determination of antimicrobial activities against bacteria, serial dilutions of the EO (0.006 to 32.0 *μ*L/mL) were prepared in Mueller-Hinton broth in 96-well microtiter plates, and for the yeast, serial dilutions of the EOs (0.006–32.0 *μ*L/mL) were prepared in 96-well microtiter plates using RPMI-1640 media buffered with morpholinopropanesulfonic acid (MOPS). 0.1 mL of the working inoculums was added to the microtiter plates, and then, they were incubated in a humid atmosphere at 37°C for 24 hours (bacteria) or 30°C for 24–48 hours (yeast). 200 *μ*L of the uninoculated medium was the sterility control (blank). Additionally, growth controls (medium with inoculums but without EO) were also included. The growth in each well was compared with the growth control well, and each test was performed in triplicate. MIC was visually determined as the lowest concentration of the EO, producing no visible growth. Furthermore, media from wells with no visible growth of bacteria on brain heart infusion agar (BHI) (Merck, Germany) were used to determine the minimum bactericidal concentration (MBC), and media from wells with no visible growth of fungi on Sabouraud dextrose agar (SDA) (Merck, Germany) were used to determine minimum fungicidal concentration (MFC). MBC and MFC were determined as the lowest concentrations yielding no more than 4 colonies that correspond to a mortality of 99.9% of the microbes in the initial inoculums [20].

### 2.4. Biofilm Preparation and Growth

Standard strains of bacteria were cultured on BHI and *C. albicans* was cultured on SDA. After 48 hours, one loop of the bacteria colonies was transferred to 20 mL of brain heart infusion broth, and one loop of the fungi colonies was transferred to 50 mL Erlenmeyer flasks containing 20 mL of Sabouraud dextrose broth (SDB) (Merck, Germany). They were incubated at 30°C on shaker at 100 rounds per minute for 24 hours. Then, the tested strains were washed twice in sterile phosphate-buffered saline (PBS) (0.8% [*w*/*v*], NaCl 0.02% [*w*/*v*], KH_2_PO_4_ 0.31% [*w*/*v*], Na_2_HPO_4_+12H_2_O 0.02% [*w*/*v*], and KCl pH 7.4); then, washed cells were resuspended in Mueller-Hinton broth (bacteria) and in RPMI 1640 buffered with MOPS (yeasts). The cell densities were adjusted to 1.0 × 10^8^ cells/mL (bacteria) and 1.0 × 10^6^ cells/mL (yeasts) at a wavelength of 530 nm. Mueller-Hinton broth and RPMI 1640 were used to add serial dilutions of *T. fallax* (0.015 to 8 *μ*L/mL) to 96-well plates. And then, the plates were incubated at 30°C for 48 hours. In addition, 200 *μ*L of the uninoculated medium was used as a negative control (blank), and each of Mueller-Hinton broth and RPMI medias with the bacteria and yeasts but without the EO was considered as positive controls [1].

### 2.5. Assessing Biofilm Formation

Plates were washed with PBS again, and biofilm formation was assessed by using a 100 *μ*L aliquot of XTT/Menadione. The plates were incubated at 37°C in a dark room for two hours. Finally, the colorimetric changes (optical density) were detected at OD_570_ nm by using a microtiter plate reader. Then, the percentage of inhibition was compared with the positive controls.

(1) $$\begin{array}{c} \text{Percentage of inhibition}:\frac{\text{OD positive control}-\text{OD experimental}}{\text{OD positive control}}. \end{array}$$

### 2.6. Qualitative Observation of Biofilm Architecture by Fluorescent Microscopy

The morphological changes in *C. albicans* biofilms treated with *T. fallax* EO (0.015 *μ*L/mL and 8 *μ*L/mL) were studied using a fluorescent microscope (BX61, Olympus, Japan). After 48 h of incubation, unattached cells were removed by washing each well three times with PBS. For 30 minutes at room temperature, adhered cells were stained with calcofluor white 0.5% (which binds to polysaccharides to outline the yeast cell walls and produces brilliant fluorescence) and observed under a fluorescent microscope. Fluorescence images were taken at 488 and 530 nm for excitation and emission, respectively [21].

### 2.7. Statistics Analysis

Descriptive data were evaluated using means and standard deviations. One-way and two-way ANOVA were used to find out the variables using SPSS version 21. *p* value of < 0.05 was considered to be statistically significant.

## 3. Results

### 3.1. GC-MS Investigation of *T. fallax* EO

**Figure 1:**
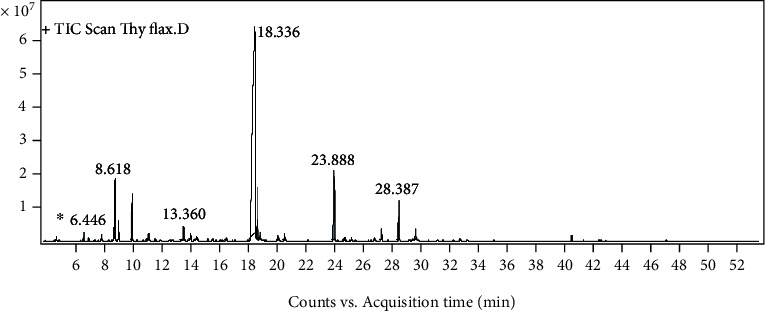
The chromatograms obtained during gas chromatography-mass spectrometry analysis.

The components of the pure EO were identified using GC/MS analyses (Figure 1). Based on the results, 20 different compounds were identified. *Thymol* was the predominant component (67.75%), followed by *E-caryophyllene* (7.27%).  Table 1 tabulates the compounds of the *T. fallax* EO.

### 3.2. Antimicrobial Activity of T. fallax EO

The antibacterial activity of the EO against the examined bacteria was determined on the basis of MIC and MBC evaluation. *T. fallax* inhibited the growth of all of the examined strains at concentrations of 0.031-16 *μ*L/mL. Throughout the study, *S. salivarius* had the lowest MICs and MBCs which were 0.031 *μ*L/mL, while *E. faecalis* had the highest (0.125, 0.250 *μ*L/mL).  Table 2 presents the MIC and MBC/MFC values of the EO towards the studied bacterial and fungal species.

### 3.3. Biofilm Formation of Microorganisms

**Figure 2:**
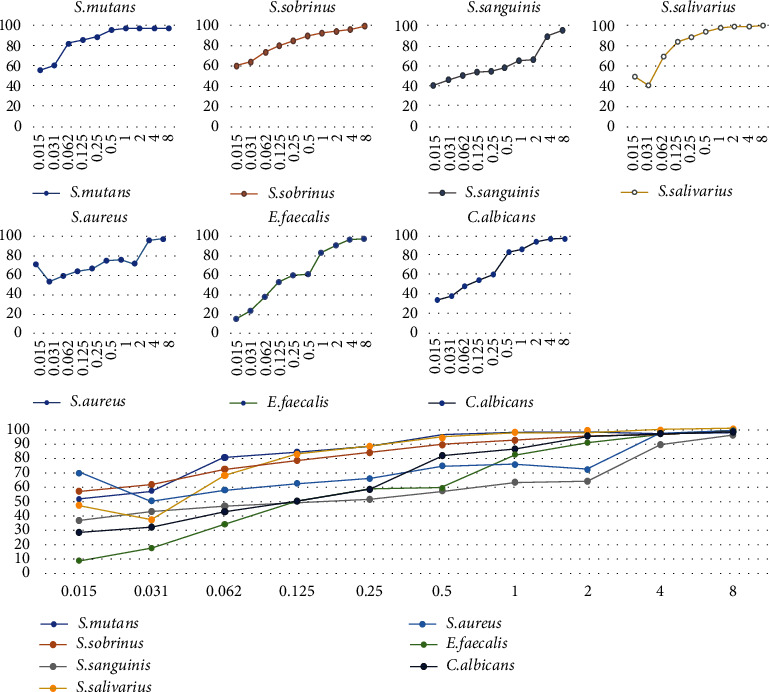
The percentage of anti-biofilm activity of the *T. fallax* EO against tested microorganisms. The *Y*-axis represents biofilm inhibition percentages, and the *X*-axis represents *T. fallax* EO concentrations.

Biofilm growth and development were assessed using the XTT reduction assay. Based on the results, *T. fallax* EO showed biofilm inhibition of greater than 95% against all of the tested microbial strains at a concentration of 8 *μ*L/mL. Indeed, *T. fallax* EO exhibited significant activity in the inhibition of biofilm formation as reflected by lower absorbance reading when compared with the untreated control. Moreover, at a concentration of 0.015 *μ*L/mL, it had biofilm inhibition of more than 50% against *S. mutans*, *S. salivarius*, and *S. aureus*. It had the highest biofilm formation inhibitory effects (*p* value < 0.05) against *S. sobrinus* and *S. salivarius* (up to 99%) and the lowest inhibitory effects against *S. sanguinis* (Figure 2).

**Figure 3:**
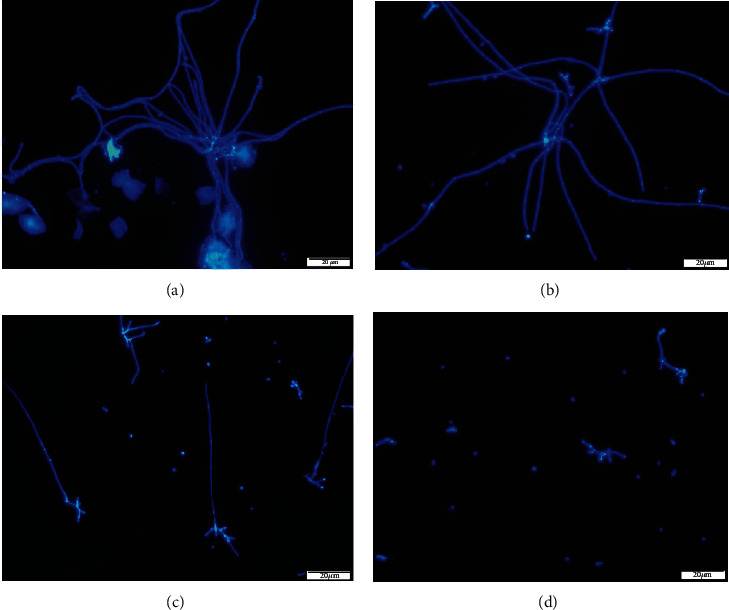
Fluorescence microscopy images of the untreated *C. albicans* biofilm formation (a and b), treated with 0.015 *μ*L/mL *T. fallax* EO (c) and 8 *μ*L/mL *T. fallax* EO (d).

As is evident, fluorescence microscopy data further validate the XTT assay results. In the control group, fluorescence microscopy images revealed mature biofilm architecture with an abundance of fungal hyphae embedded within the extracellular matrix (Figures 3(a) and 3(b)). Following *T. fallax* EO treatment, the biofilm structure was destroyed, the extracellular matrix was reduced, mycelia were shortened, and only a small amount of biofilm remained (Figures 3(a) and 3(d)). These results confirmed that *T. fallax* EO significantly affected the biofilm structure of *C. albicans.*

## 4. Discussion

As mentioned, medicinal plants are now widely used due to their various properties. The genus *Thymus* L. is a member of the *Lamiaceae* family, a famous aromatic herb, which is widely grown in the Mediterranean region [22]. *T. fallax* is one of the 14 *Thymus* species in Iran, and its EO can be extracted from its leaves and flowering tops [23]. The major compounds detected in the GC/MS results were *thymol* (67.4%), *caryophyllene* (7.23%), and c*arvacrol* (4.40%). These results were almost similar to other studies in Iran which were characterized by a high content of *thymol* (61.14-79.74%) [24–26]. In another investigation in Turkey, *thymol* with a lesser amount in comparison with the current study (41.48%) was the main constituent of the oil [27]. On the other hand, some other investigations in both Turkey and Iran showed that *carvacrol* was the principal component (66.1-69.2%) and *thymol* amounts were much lower in comparison with our results [17, 28, 29]. The EO content and the difference between GC/MS results may be affected by the time of harvest as well as environmental factors such as climate and soil condition. The EO successfully inhibited the growth of all tested bacteria and fungi. Even *E. faecalis*, which is naturally resistant to many antibiotics, was inhibited at MICs in 0.031-0.125 *μ*L/mL range, which is lower than MICs of another *Thymus* species [29]. *T. fontanesii*, with similar major constituents (*thymol* = 29.3%), inhibited the growth of *S. aureus* and *C. albicans* at the MIC of 1 *μ*L/mL [30]. In comparison to other EOs, the MIC of *T. fallax* was lower than other EOs in the previous studies such as *lavender*, *thyme*, *peppermint,* and *saliva* (1-64 *μ*L/mL) [31–33]. Generally, *T. fallax* showed strong antibacterial activity in comparison with others [30–34]. These differences might be due to various amounts of monoterpenes and the purity of EO.

Similarly, Küçükbay et al. studied the antimicrobial activities of *T. fallax* EO by the disc diffusion method and found moderate inhibitory activity against *S. aureus* and *C. albicans.* The lower inhibitory activities in their study in comparison to ours might be due to the different *thymol/carvacrol* ratio [17]. In another experiment using the disk diffusion test by Ozturk and Ercisli, *T. fallax* had considerable antibacterial activity against other bacteria at a concentration of 31.25-500 *μ*g/mL [35].

*Thymol* is an oxygenated monoterpene, which its phenolic part can dissolve through the bacterial cell membrane and mitochondria and then interfere with their normal arrangement and metabolic mechanisms [36]. The mentioned mode of action is similar to the chlorhexidine antibacterial mechanism. Nevertheless, the antibacterial activity of *T. fallax* is not just due to one specific mechanism or compound, and other mechanisms are also considered for its antibacterial activity. However, the above-mentioned mechanism is more approved. *Carvacrol*, another component of the EO, is very similar to thymol functionally and structurally [37] *E-Caryophyllene* is also known for its anti-inflammatory and antibacterial properties [38].

Regarding the impact of the EO on the biofilm growth, which is shown in Figure 2, it can be noticed that the applied treatment caused a huge reduction in biofilm formation of the examined strains. The results showed that the effects of higher concentrations of EO led to a significantly greater cutback in biofilm growth. Based on the results, *T. fallax* showed a great anti-biofilm activity. Other studies on EOs containing *thymol* also suggested that this monoterpene can significantly reduce the biofilm formation by *S. mutans*, methicillin-resistant *S. aureus*, and *E. faecalis* [39–41]. Interestingly, Miladi et al. also noticed that *thymol* can synergistically increase the antibiotic effect of tetracycline against oral strains [40]. Dalleau et al.'s investigations about treatment of *C. albicans* biofilms with *thymol* (0.06%) resulted in more than 80% inhibition [42]. In another study by Kerekes et al.'s scanning electron microscopy was used to investigate the structural differences between *S .aureus* biofilms before and after treatment with *thyme* EO and *thymol* (0.1 mg/mL). After the treatment, the three-dimensional structure embedded in the extracellular polysaccharide layer appeared only in microcolonies with a simple shape, less attached cells, and more damaged [43]. So far, no other investigations have been done into the anti-biofilm activity of *T. fallax* against oral bacteria; therefore, the results were inevitably compared with other EOs with similar compounds. It is likely that the greater or lesser efficiency of EO's contributes to the amount of composition and the time of exposure. In addition, it is noticeable that biofilms are well known for having considerable structural and physiological heterogeneity, even for those made by the same microorganisms under various environmental situations. In this study, both the anti-biofilm and antimicrobial activities of *T. fallax* against infectious oral bacteria and *Candida* were examined. Even though these effects were investigated on highly resistant and intracanal bacteria, we recommend using *T. fallax* EO as a mouth rinse or in vivo investigations in further studies to achieve more reliable results.

## 5. Conclusion

The current study revealed *T. fallax* possess considerable antimicrobial and anti-biofilm activities due to its major phenolic components, indicating that the EO is capable of preventing and interfering with microorganisms causing oral infections. Additionally, in addition to its antibacterial properties, it has a pleasant smell and flavor. *T. fallax* can be employed in oral hygiene products and may have a promising future in the treatment of oral bacteria.

## Figures and Tables

**Table 1:** The composition of *T. fallax* EO obtained by gas chromatography-mass spectrometry.

Peak	Compound	RT^a^	Area = 100^b^	KI (calculated)^c^
1	*Pinene (a-)*	6.446	0.440	930.4500978
2	*Myrcene*	7.699	0.440	979.4911937
3	*Cymene (ρ-)*	8.618	3.956	1011.880597
4	*Cineole (1,8)*	8.888	1.456	1019.940299
5	*Terpinene (γ)*	9.799	3.292	1047.134328
6	*Terpinolene*	10.971	0.582	1082.119403
7	*Isoborneol*	13.36	1.571	1146.651029
8	*Terpineol (trans-β-)*	13.846	0.806	1159.317175
9	*Terpineol*	14.306	0.474	1171.305708
10	*Thymol, methyl ether*	16.334	0.298	1223.076923
11	*Thymol*	18.336	67.755	1272.915111
12	*Carvacrol*	18.525	4.424	1277.620115
13	*Guaiacol (ρ-vinyl-)*	18.732	0.697	1282.773214
14	*Eugenol*	20.436	0.691	1325.336658
15	*Caryophyllene (E-)*	23.888	7.276	1411.843807
16	*Bisabolene (β-)*	27.17	1.104	1496.7158
17	*Unknown*	28.387	3.624	1529.299191
18	*Atlantol (β-)*	29.558	1.104	1570.862534

^a^Retention index. ^b^The amount of each compound was calculated based on the total essential oil chromatogram area peak. ^c^Kovacs index values present the retention indices calculated against C_6_-C_24_ n-alkanes on the mentioned column.

**Table 2:** Antimicrobial effects of *T. fallax* EO on the bacteria and yeast strains based on broth microdilution method.

Species	ATCC^a^	MIC^b^ (*μ*L/mL)	MBC^c^/MFC^d^ (*μ*L/mL)
*S. mutans*	35668	0.031	0.062
*S. sobrinus*	27607	0.031	0.125
*S. sanguinis*	10556	0.031	0.125
*S. salivarius*	9222	0.031	0.031
*S. aureus*	52268	0.062	0.125
*E. faecalis*	51299	0.125	0.250
*C. albicans*	10261	0.06	0.125

^a^ATCC: American Type Culture Collection. ^b^Minimum inhibitory concentration. ^c^Minimum bactericidal concentration. ^d^Minimum fungicidal concentration.

## Data Availability

The data used to support the findings of this study were supplied by the Shiraz University of Medical Sciences under license and so cannot be made freely available. Requests for access to these data should be made to Kamiar Zomorodian (zomorodian@sums.ac.ir or kzomorodian@gmail.com).
