# Corrigendum to “Antimicrobial and Anti-Biofilm Activities of *Thymus fallax* Essential Oil against Oral Pathogens”

**DOI:** 10.1155/bmri/9797835

**Published:** 2024-12-13

**Authors:** Maryam Moshaverinia, Sarina Sahmeddini, Fatemeh Lavaee, Zahra Zareshahrabadi, Kamiar Zomorodian

**Affiliations:** ^1^Department of Oral and Maxillofacial Disease, School of Dentistry, Shiraz University of Medical Sciences, Shiraz, Iran; ^2^Student Research Committee, Shiraz University of Medical Sciences, Shiraz, Iran; ^3^Oral and Dental Disease Research Center, Oral and Maxillofacial Disease Department, School of Dentistry, Shiraz University of Medical Sciences, Shiraz, Iran; ^4^Basic Sciences in Infectious Diseases Research Center, Shiraz University of Medical Sciences, Shiraz, Iran; ^5^Department of Medical Mycology and Parasitology, School of Medicine, Shiraz University of Medical Sciences, Shiraz, Iran

In the article titled “Antimicrobial and Anti-Biofilm Activities of *Thymus fallax* Essential Oil against Oral Pathogens” [1], there was a spelling error in author Fatemeh Lavaee's name in the author list, where Fatemeh Lavee should have read Fatemeh Lavaee.
